# Successful EUS-guided fine-needle biopsy using a forward-viewing echoendoscope for local recurrence at the choledochojejunal anastomotic site 13 years after pancreaticoduodenectomy for cholangiocarcinoma

**DOI:** 10.1097/eus.0000000000000096

**Published:** 2024-12-30

**Authors:** Akihiko Kida, Jun Asai, Tatsuya Yamashita, Takeshi Urabe, Taro Yamashita

**Affiliations:** 1 Department of Gastroenterology, Public Central Hospital of Matto Ishikawa, Hakusan, Japan; 2 Department of Gastroenterology, Kanazawa University Hospital, Kanazawa, Japan.

Thirteen years after subtotal stomach-preserving pancreaticoduodenectomy with modified Child reconstruction for distal cholangiocarcinoma (dCCA), an 83-year-old man presented with jaundice. Blood testing revealed cholangitis. Dilated intrahepatic and remaining common bile ducts and a 23-mm irregular intra-abdominal mass at the choledochojejunal anastomotic site (CAS) were detected on computed tomography [Figure 1]. Positron emission tomography–computed tomography showed fluorodeoxyglucose uptake in this lesion [Figure 2]. Therefore, obstructive jaundice due to local recurrence after surgery for dCCA was suspected. However, because surgery was performed 13 years ago, local recurrence was not expected. A definitive diagnosis of local recurrence and endoscopic biliary stenting were planned. A single-balloon endoscope (SIF-H290S; Olympus Medical Systems, Tokyo, Japan) with a sliding tube was reached to the CAS. Anastomotic stenosis with granular mucosa was observed [Figure 3] and biopsied. Endoscopic biliary stenting was performed with 2 plastic stents and a fully covered self-expandable metal stent at the CAS. Biopsy samples were negative for malignancy. Percutaneous transhepatic tumor biopsy by abdominal ultrasound and EUS-guided fine-needle biopsy (EUS-FNB) using a transgastric approach were planned; however, difficulties were associated with delineating the lesion. Therefore, EUS-FNB using a forward-viewing echoendoscope (FV-echoendoscope) (TGF-UC260J; Olympus Medical Systems) was planned. FV-echoendoscope was safely reached to the CAS [Figure 4]. EUS was conducted at the CAS using fully covered self-expandable metal stent as an indicator [Figure 5] and showed a 23-mm irregular hypoechoic lesion [Figure 6]. EUS-FNB was performed [Figure 7] and showed adenocarcinoma [Figure 8]. Therefore, local recurrence after surgery for dCCA was diagnosed and treated with radiation.

**Figure 1:**
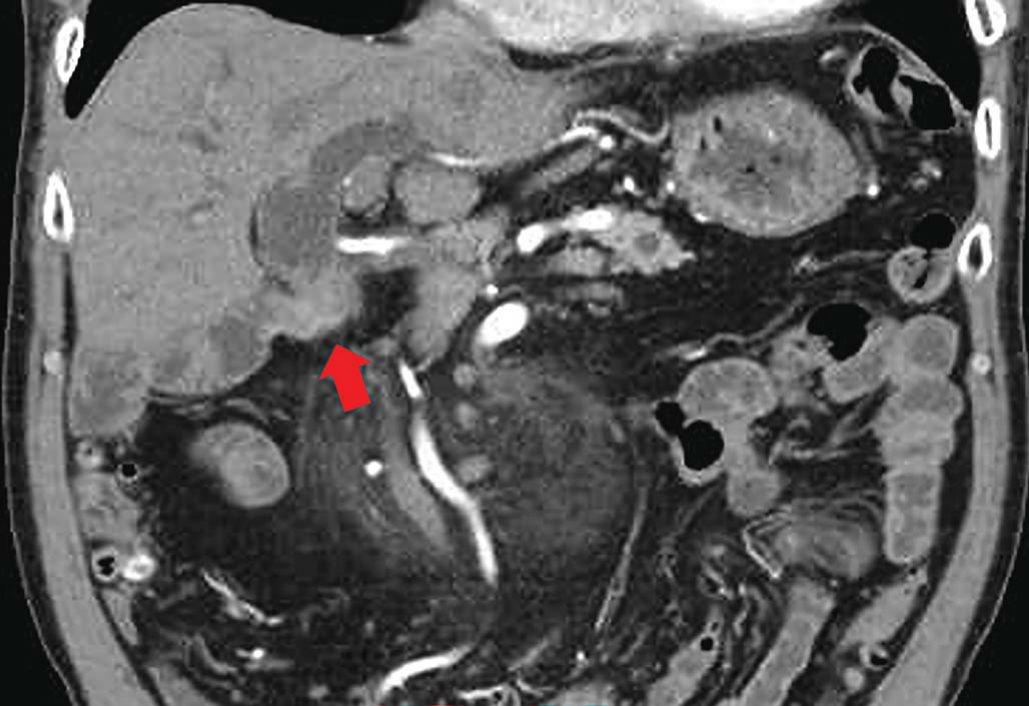
Dilated intrahepatic and remaining common bile ducts and a 23-mm irregular intra-abdominal mass at the choledochojejunal anastomotic site (CAS) were detected on computed tomography (CT). A red arrow shows a 23-mm irregular intra-abdominal mass at the CAS.

**Figure 2:**
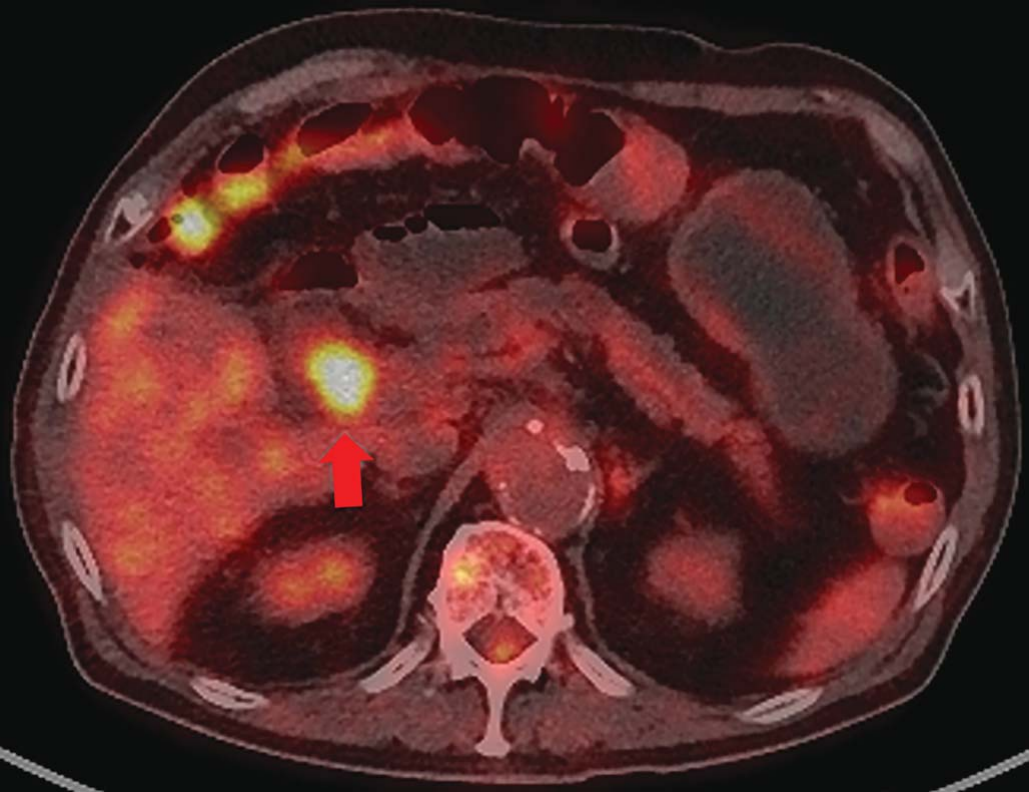
Positron emission tomography–computed tomography shows fluorodeoxyglucose uptake in the intra-abdominal mass at the choledochojejunal anastomotic site (CAS). A red arrow shows an intra-abdominal mass at the CAS with fluorodeoxyglucose uptake.

**Figure 3:**
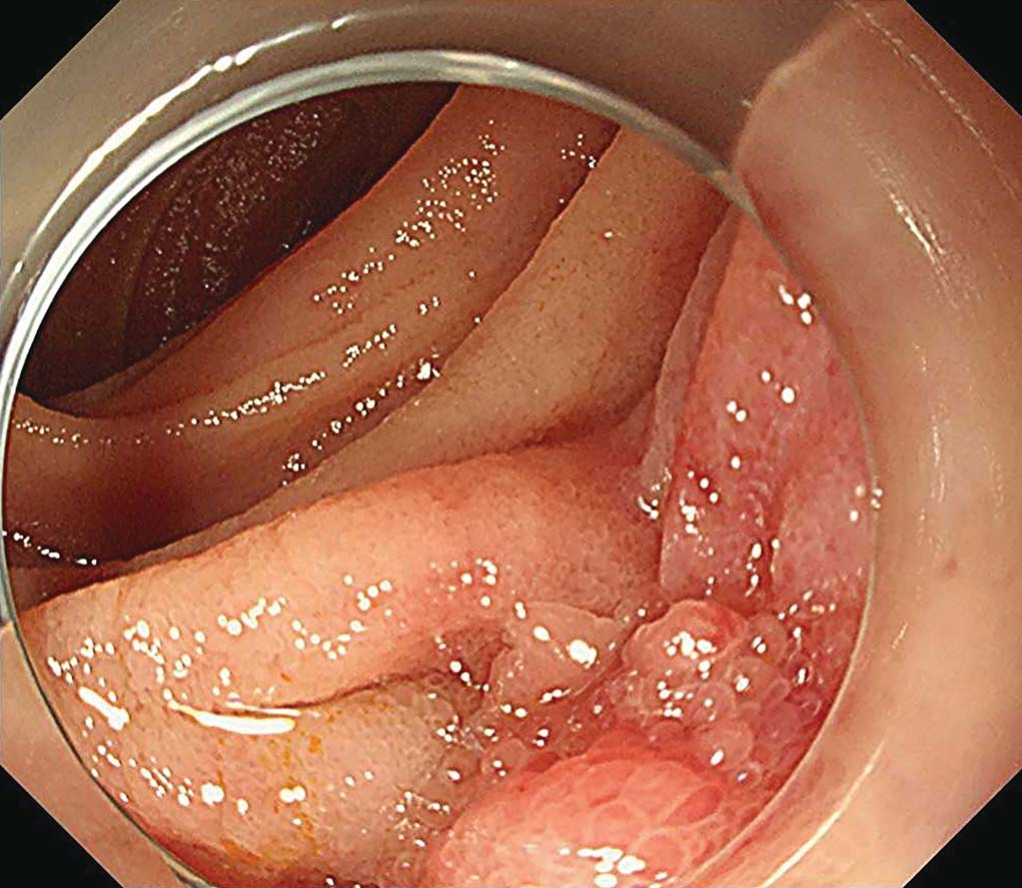
Anastomotic stenosis with granular mucosa was observed at the choledochojejunal anastomotic site (CAS).

**Figure 4:**
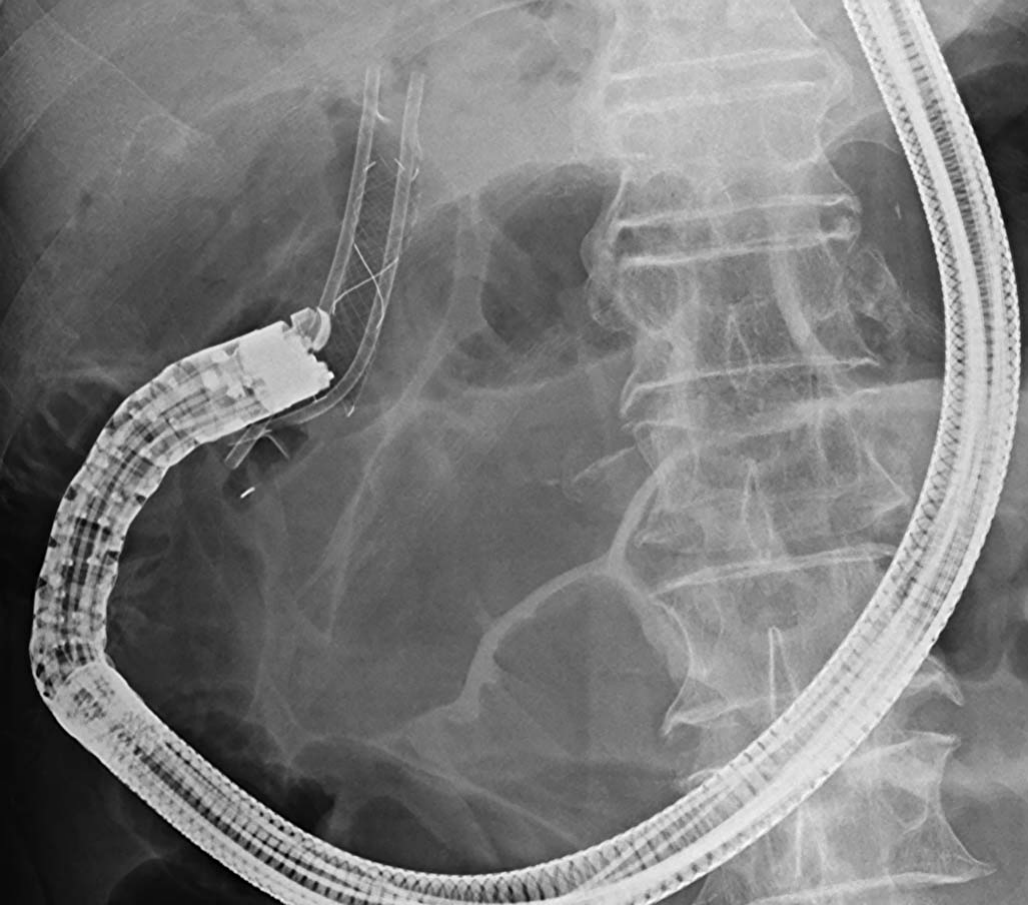
A forward-viewing echoendoscope was safely reached to the choledochojejunal anastomotic site (CAS).

**Figure 5:**
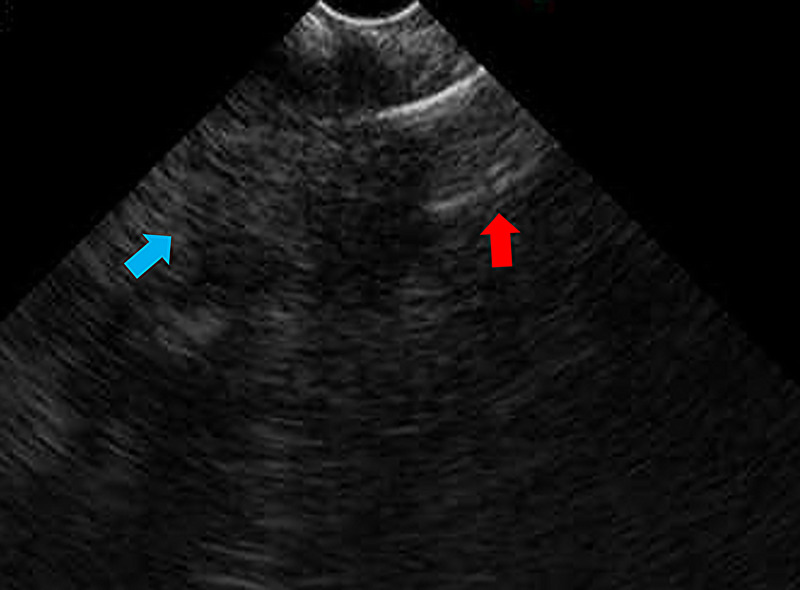
EUS was conducted at the choledochojejunal anastomotic site (CAS) using fully covered self-expandable metal stent (FCSEMS) as an indicator. A red arrow shows a FCSEMS. A blue arrow shows a part of hypoechoic lesion that intersected the FCSEMS.

**Figure 6:**
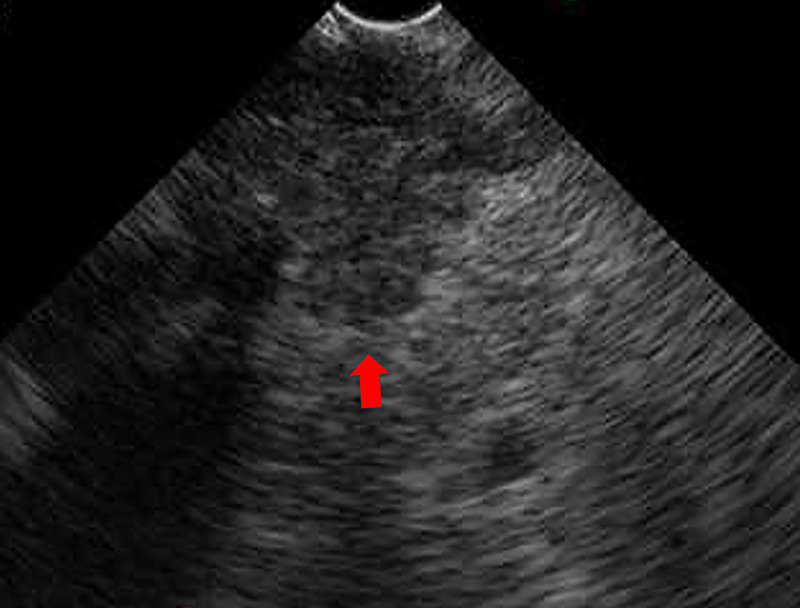
EUS shows a 23-mm irregular hypoechoic lesion. A red arrow shows a 23-mm irregular hypoechoic lesion.

**Figure 7:**
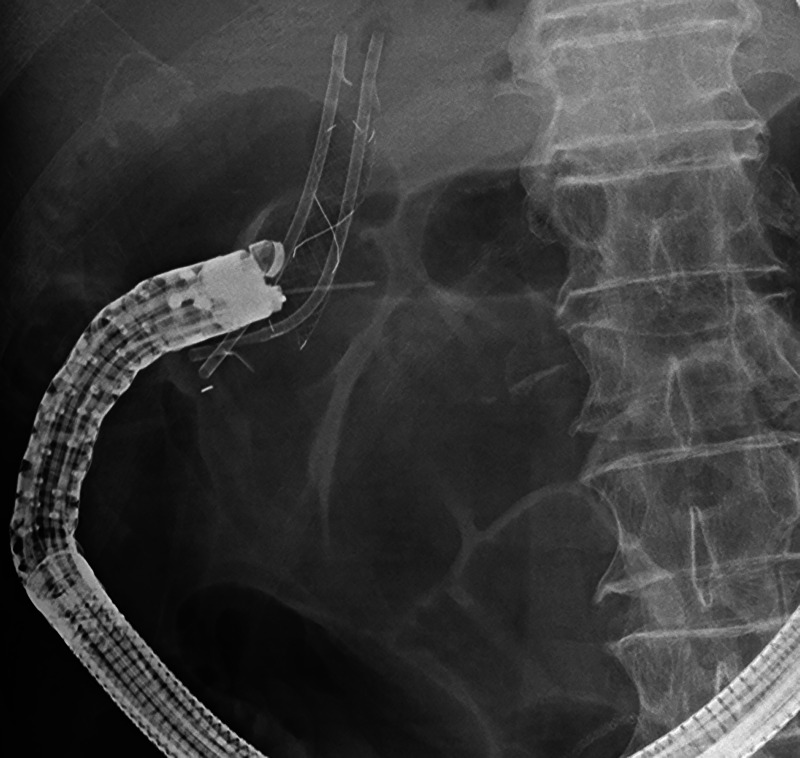
EUS-guided fine-needle biopsy (EUS-FNB) was performed for an intra-abdominal mass at the choledochojejunal anastomotic site (CAS).

**Figure 8:**
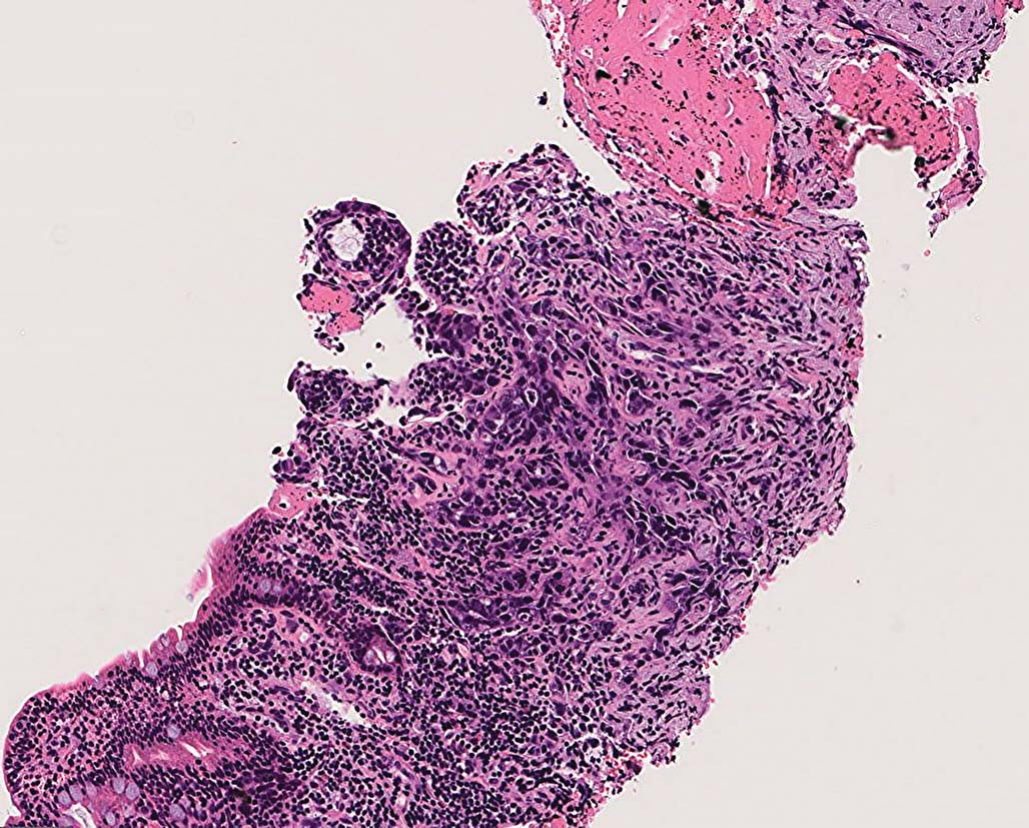
EUS-guided fine-needle biopsy (EUS-FNB) shows adenocarcinoma.

Local recurrence after surgery for dCCA generally occurs within 10 months.^[1]^ Local recurrence after 13 years, as in the present case, is extremely rare. The usefulness of FV-echoendoscope-biliary drainage for choledochojejunal anastomotic stenosis has been demonstrated^[2]^; however, that of EUS-FNB using FV-echoendoscope for local recurrence at the CAS remains unclear. Although EUS-FNB for biliopancreatic diseases in cases with a surgically altered anatomy may be possible with an oblique-viewing echoendoscope, only FV-echoendoscope may reach the target site in some cases.^[3]^ Therefore, EUS-FNB using FV-echoendoscope may be useful on cases for which a tissue diagnosis is difficult, such as the present case.

## Data Availability

All data relevant to the case are included in the article.
